# Femoral nerve block-sciatic nerve block vs. femoral nerve block-local infiltration analgesia for total knee arthroplasty: a randomized controlled trial

**DOI:** 10.1186/s12871-015-0160-3

**Published:** 2015-12-15

**Authors:** Mari Nagafuchi, Tomoyuki Sato, Takahiro Sakuma, Akemi Uematsu, Hiromasa Hayashi, Hidenori Tanikawa, Kazunari Okuma, Akira Hashiuchi, Junya Oshida, Hiroshi Morisaki

**Affiliations:** 1 Department of Anesthesiology, Saitama City Hospital, Mimuro 2460, Saitama Midori-ku, Saitama 336-8522 Japan; 2 Department of Orthopedics, Saitama City Hospital, Mimuro 2460, Saitama Midori-ku, Saitama 336-8522 Japan; 3 Department of Anesthesiology, School of Medicine, Keio University, Shinanomati 35, Shinjuku-ku, Tokyo, 160-0016 Japan

**Keywords:** Postoperative pain, Knee arthroplasty, Nerve block, Femoral nerve, Sciatic nerve, Local anesthesia

## Abstract

**Background:**

The use of femoral nerve block (FNB) combined with sciatic nerve block (SNB) after total knee arthroplasty (TKA) has recently become controversial. Local infiltration analgesia (LIA) has been reported to be effective for postoperative TKA pain control. We aimed to assess whether LIA with continuous FNB is as effective as SNB combined with continuous FNB.

**Methods:**

This was a prospective, randomized, single-center, observer-blinded, parallel group comparison trial of 34 American Society of Anesthesiologists (ASA) physical status 1–3 patients who underwent TKA and fulfilled the inclusion and exclusion criteria. Patients were randomized into two groups: a periarticular LIA and FNB group (group L, *n* = 17), and an SNB and FNB group (group S, *n* = 17). In both groups, participants received FNB with 20 mL of 0.375 % ropivacaine, and 5 mL h^−1^ of 0.2 % ropivacaine after surgery. In group L, participants received 100-ml injections of 0.2 % ropivacaine and 0.5 mg epinephrine to the surgical region. In group S, participants received SNB with 20 ml of 0.375 % ropivacaine.

After TKA, Numeric Rating Scale (NRS) scores for the first 24 h post-operation were compared via repeated-measures analysis of variance (ANOVA) as the primary outcome. Other outcome measures included NRS score changes within groups, area under the curve for the NRS scores, total analgesic dose, change in knee flexion and extension, pain control satisfaction, nausea and vomiting, and hospital stay duration.

**Results:**

NRS score changes were greater in group L than in group S (*P* < 0.01, ANOVA) and greater in group L than in group S at three postoperative time points: 3 h (*P* < 0.01), 6 h (*P* < 0.01), and 12 h (*P* = 0.013; Mann–Whitney *U* test). Changes in the mean NRS score were observed in each group (*P* < 0.01, Friedman test). No significant differences were detected in the other outcome measures (Mann–Whitney *U*, Wilcoxon signed-rank, and chi-squared tests).

**Conclusions:**

Sciatic nerve block with femoral nerve block is superior to local anesthetic infiltration with femoral nerve block for postoperative pain control within 3–12 h of total knee arthroplasty.

**Trial registration:**

UMIN-CTRID:000013364R:000015591

## Background

Total knee arthroplasty (TKA) is a common operative procedure performed to improve mobility and quality of life [1]. Femoral nerve block (FNB) was recently reported to be useful for postoperative pain control after TKA [2, 3]. FNB can be performed more safely than epidural block in the presence of an anticoagulant [4, 5]. In contrast, continuous FNB with sciatic nerve block (SNB) was found to reduce analgesic requirements after TKA [6]. The pain and discomfort in the posterior aspect of the knee associated with TKA were reported to be reduced by SNB, and a single SNB injection was found to reduce severe pain on the day of surgery [7]. We have thus used continuous FNB combined with an SNB procedure for several years for postoperative pain control after TKA.

However, the benefit of adding SNB to FNB to improve analgesia after TKA has recently become controversial [8]. Additionally, it has been reported that local infiltration analgesia (LIA) is superior to both FNB and epidural anesthesia for postoperative TKA pain control [9–11]. LIA is also reported to reduce pain in the early postoperative period [12–17]. The objective of this study was to compare the analgesic effect of LIA with that of SNB in combination with a continuous FNB for postoperative pain control after TKA. Our null hypothesis was that there would be no difference in NRS score changes between the groups.

## Methods

This prospective, single-center, randomized, observer-blinded, parallel group comparison study was conducted in accordance with the ethical principles of the Helsinki Declaration, and was approved (authorization number A2405) by the Regional Ethical Committee of Saitama City Hospital (Research Ethics Committee No. 11000176) in September 2012. The registry number of this RCT is UMIN-CTR ID: 000013364 R: 000015591. The patients were recruited from October 2012 to July 2013. Thirty-four subjects were randomly assigned to two groups: an SNB group (group S) or a local anesthesia group (group L). All subjects provided written informed consent to participate in this study. Anesthesiologists who did not perform the anesthesia enrolled the participants and assigned them to the groups. The allocation ratio was 1:1. In total, 34 numbered cards assigned as L1-L17 and S1-S17 in an opaque envelope were used for random allocation to groups. The envelope was concealed in a safe and was opened on the morning of the operation. The patients and evaluators were blinded to the group assignments throughout the study.

The inclusion criteria were as follows: Scheduled for unilateral TKA for degenerative arthritis, American Society of Anesthesiologists (ASA) physical status 1–3, weight 40–99 kg, and fully able to understand the study contents from oral and written descriptions. The exclusion criteria were: Scheduled for bilateral TKA, regular narcotic use, allergies to any study drug, neuromuscular disease, sensory disturbances of the legs, severe diabetes, heart failure (New York Heart Association classification greater than 2), renal impairment with an estimated glomerular filtration rate of < 60 mL min^−1^ 1.73 m^−2^ or liver dysfunction (Child–Pugh classification greater than class B), and inability to be assessed via the Numeric Rating Scale (NRS). All patients received instruction in the use of the NRS, where the number 0 on the ruler represented the absence of pain and the number 10 represented the most severe pain they had ever experienced, and they were asked to indicate the number that best reflected their pain on that scale.

The same team of orthopedists performed all surgeries, and the same anesthesiologist performed the anesthesia for all cases. All patients underwent general anesthesia induced by 2 mg kg^−1^ propofol and 50 μg of fentanyl, followed by insertion of a laryngeal airway. General anesthesia was maintained with inhaled 1–2 % sevoflurane in oxygen and air. Additionally, remifentanil administration was started at incision from 0.1 μg kg^−1^ min^−1^, increased or decreased when the blood pressure or heart rate changed by approximately 10 %, and stopped at least prior to skin closure. No additional narcotics or analgesics including fentanyl were administered during the operation.

The operation was performed using the midvastus approach. None of the participants underwent patellar resurfacing. The resurfacing criterion in our facility is International Cartilage Repair Society Grade IV. The implant models used in this study were of the Triathlon (Stryker, Mahwah, NJ, USA) or Verilast (Smith & Nephew, Andover, MA, USA) type. The implant was fixed with cement (Simplex P Bone Cement with cefazolin) on the cut bone surfaces after confirmation of the joint gap, and balanced by trial and error. A vacuum drain was not used. Edoxaban tosilate hydrate (Lixiana®, Daiichi-Sankyo, Tokyo, Japan; 30 mg) was administered orally for 10 days from postoperative day 2.

### Group S

Subjects were moved to a lateral recumbent position after insertion of the laryngeal airway. An ultrasound-guided (M-Turbo Fujifilm SonoSite, Tokyo, Japan) SNB approach was utilized in combination with a 0.5–1.0 mA nerve stimulus for 0.1 ms (Stimplex HNS12, B. Braun, Melsungen, Germany). By setting the echo-probe (C60x/5-2 MHz Transducer Fujifilm SonoSite, Tokyo, Japan) parallel to the line created by the posterior superior iliac spine and ischial tuberosity, we inserted a needle (Stimplex D Needle STD-2280, B. Braun, Melsungen, Germany) with an in-plane approach from the lateral aspect of the ultrasound probe and injected 20 mL of 0.375 % ropivacaine after the position of the needle tip was confirmed by a motor response from the ankle.

After SNB, the patient was moved into the supine position, and ultrasound-guided FNB was performed in combination with 0.5–1.0 mA nerve stimulation for 0.1 ms (Stimplex HNS12, B. Braun, Melsungen, Germany). We placed the echo-probe (HFL38x/13-6 MHz Transducer, Fujifilm SonoSite, Tokyo, Japan) parallel to the inguinal ligament region to identify the femoral nerve and then inserted a needle (Contiplex Touphy B. Braun, Melsungen, Germany) using an in-plane approach from the lateral aspect of the ultrasound probe. We then injected 20 mL of 0.375 % ropivacaine after the position was confirmed by contraction of the quadriceps muscle. After the ropivacaine bolus injection, a catheter tip (Aesculap, B. Braun, Melsungen, Germany) was placed 3 cm from the needle tip. A continuous FNB (5 mL h^−1^) of 0.2 % ropivacaine was commenced at the end of the operation.

### Group L

Subjects in group L underwent FNB similarly to those in group S. The local anesthetic mixture was prepared from 100 mL of 0.2 % ropivacaine by adding 0.5 mL (0.5 mg) of adrenaline. The local anesthetic mixture was administered three times in measured doses as follows:

1. 20 mL of the local anesthetic mixture was administered intracutaneously to the surgical region at the start of the operation.
2. 50 mL of the local anesthetic mixture was administered as infiltration anesthesia to sites posterior to the articular capsule near the incised part, namely the vastus intermedius, vastus lateralis, and lateral collateral ligament, before the injection of cement.
3. 30 mL of the local anesthetic mixture was administered intraarticularly at the end of the operation.

A continuous femoral block of 0.2 % ropivacaine at 5 mL h^−1^ was commenced at the end of the operation, as in group S.

The participants were administered a 25-mg diclofenac suppository if their NRS score was > 3 upon exiting the operating room, and if at 3, 6, 12, and 24 h after that time or at any other time they requested an analgesic and had an NRS score higher than 3. Repeated administrations of 25-mg diclofenac suppositories were allowed after a 3-h interval. If this method was inadequate and the patient could not cope with the pain (NRS score > 3), 15 mg of pentazocine was injected intramuscularly. We did not apply a strict fast-track recovery program in this study.

### Outcome measures

The primary outcome was the change in NRS scores between groups L and S at five time points: upon exiting the operating room and 3, 6, 12, and 24 h later. Nurses in the operating room and on the ward who were blinded to the group allocations recorded the NRS scores.

Other outcome measures included NRS score changes within groups, the area under the curve of the NRS scores, total dose of diclofenac, change in knee flexion and extension, pain control satisfaction, nausea and vomiting, and hospital stay duration.

Physiotherapists measured knee flexion and extension using an angle meter every postoperative day. The Knee Society Score (The 2011 Knee Society Knee Scoring System) [18] was recorded before the operation and at discharge. Furthermore, the participants were asked about numbness or unusual sensations in their tongue and mouth, metallic tastes, dizziness, tinnitus, and agitation, and they were also checked for possible ropivacaine side effects by evaluating their level of consciousness and convulsions upon exiting the operating room and at 30 min, 1 h, and 3 h after that time. Nausea and vomiting were evaluated at the same time by inquiring about the presence or absence of symptoms. Nurses on the ward who were blinded to the group allocations checked these items. In addition, during the first 24 h after the operation, the nurses and physicians in charge observed the participants closely on the ward every 4 h. If symptoms of ropivacaine toxicity were detected, the continuous infusion of ropivacaine was discontinued, and symptomatic treatment was performed accordingly. In cases of unanticipated side effects, the details were recorded.

Analgesic administration times were recorded, and the total doses were calculated. On the third postoperative day, a survey was administered relating to pain control satisfaction, consisting of a five-point scale (1 = extremely severe pain; 2 = quite severe pain; 3 = neutral; 4 = generally content; 5 = extremely content). No changes to trial outcomes or methods occurred after the trial commenced.

### Statistics

Repeated measures analysis of variance (ANOVA) was used to compare the changes in NRS scores between groups S and L. The differences in NRS scores at each time point and the area under the curve of the NRS scores were analyzed using the Mann–Whitney *U* test. The changes in NRS scores within each group were analyzed by the Friedman test. The values at 3, 6, 12, and 24 h were compared to those at 0 h by the Wilcoxon signed-rank test with Bonferroni correction in both groups, after differences within groups were confirmed. The *P* value for the primary outcome measure was determined using a two-tailed test. Other ordinal variables and continuous variables were analyzed by the Mann–Whitney *U* test or Wilcoxon signed-rank test. Categorical variables were analyzed by a chi-squared test for independence.

Continuous variables are presented as the mean and standard deviation. Ordinal variables are presented as the median and interquartile range. Categorical variables are presented as percentages.

The sample size of the primary outcome measure was calculated by G*Power 3 (Erdfelder & Buchner, 2007). With an alpha error of 0.05, a beta error of 0.2, and an effect size, f, of 0.25, the required sample size for detecting a significant difference between the groups was calculated to be 28. We enrolled 34 cases (17 to each group), anticipating that some patients may be lost to follow-up. Statistical analyses were performed using SPSS (SPSS Statistics 19 for Windows; SPSS Inc, Chicago, IL). Significance was defined as *P* < 0.05.

## Results

### Randomized trial progress and participant background

**Fig. 1:**
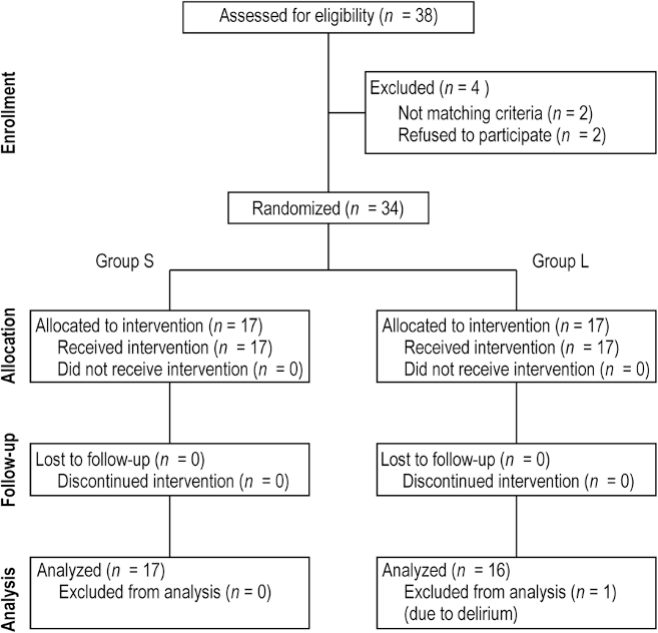
Flow diagram generated in accordance with CONSORT 2010 guidelines

Thirty-eight patients were assessed as competent. Four patients were excluded, and 34 participants were randomized into the study groups. Seventeen participants were allocated to each group. In group L, one participant was excluded after allocation, as an NRS score could not be obtained because of delirium. A patient flow diagram generated in accordance with the CONSORT 2010 statement guidelines is shown in Fig. 1.

No hospital readmissions or infections occurred during the first 60 postoperative days.

The patients’ characteristics were similar in both groups. No significant difference was detected in side, sex, age, weight, body mass index, ASA status, operation time, anesthetic time, intraoperative blood loss, intraoperative fluids, remifentanil dosage, implant choice, knee flexion and extension, or Knee Society Score (Table 1).

**Table 1:** Patient characteristics and clinical variables

	Group L	Group S	*P* value
	(*n* = 16)	(*n* = 17)	
Side, right : left, *n* (%)	10 : 6 (63 : 38)	8 : 9 (47 : 53)	0.37
Sex, men : women, *n* (%)	3 : 13 (19 : 81)	2 : 15 (12 : 88)	0.58
Age, years (range)	73 (5.9)	72 (10)	0.97
Weight, kg (range)	62 (12.5)	55 (8.2)	0.14
Body mass index (range)	27 (5.0)	25 (3.5)	0.10
ASA status 1/2/3	2 (0)	2 (0)	0.27
Surgical time (min) (range)	81 (20)	71 (15)	0.09
Anesthetic time (min) (range)	159 (25)	147 (19)	0.09
ROM (preoperative extension) (degrees)	10 (9.6)	8.4 (8.5)	0.65
ROM (preoperative flexion) (degrees)	122 (6.7)	128 (13)	0.08
KSS (score 0–100)	36 (20)	35.5 (19)	0.69
Intraoperative blood loss (mL)	3.5 (12)	20 (36)	0.14
Intraoperative fluids (mL)	1156 (210)	1309 (231)	0.06
Remifentanil dosage (mcg)	148 (45)	117 (52)	0.08
Implant T : V, *n* (%)	5 : 11 (31 : 69)	4 : 13 (24 : 76)	0.62

Data are expressed as the mean (SD), median (interquartile range: value at 75 %–value at 25 %), or n (%), and were compared using the Mann–Whitney *U* test or chi-squared test

*T* Triathlon, *V* Verilast, *KSS* Knee Society Score

### Postoperative outcomes

**Fig. 2:**
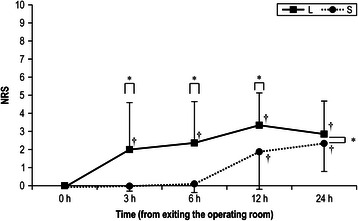
Changes in NRS scores from 0–24 h in groups L and S after TKA. The groups were compared by repeated-measures analysis of variance and Mann–Whitney U tests. Comparisons within the groups were analyzed by the Friedman test and Wilcoxon signed-rank test with Bonferroni correction (versus the 0-h value). The mean and SD are indicated. ^*^P < 0.05, comparisons between the groups; ^†^*P* < 0.05, comparisons within the groups. NRS, numerical rating scale; TKA, total knee arthroplasty

A significant difference was detected in NRS score changes between groups S and L (*P* < 0.01). The NRS scores were higher in group L than in group S at three time points, i.e., 3 h (*P* < 0.01), 6 h (*P* < 0.01), and 12 h (*P* = 0.013) after patients exited the operating room (Fig. 2). Significant NRS score changes were apparent within each group. Significant changes from the mean NRS score at 0 h were recorded at 12 h (*P* < 0.01) and 24 h (*P* < 0.01) in group S, and at 3 h (*P* = 0.011), 6 h (*P* < 0.01), 12 h (*P* < 0.01), and 24 h (*P* < 0.01) in group L after the patients exited the operating room (Fig. 2). A significant difference in the area under the curve for the NRS scores was detected between the groups (Table 2).

**Table 2:** Comparison of NRS scores and area under the curve of NRS scores between the groups

Time after exiting room	Group L	Group S	*P* value
	(*n* = 16)	(*n* = 17)	
0 h	0 ± 0	0 ± 0	
3 h	2.13 ± 2.66	0.06 ± 0.24	<0.01
6 h	2.5 ± 2.34	0.18 ± 0.39	<0.01
12 h	3.5 ± 1.83	2 ± 2.12	0.013
24 h	3 ± 1.86	2.47 ± 1.59	0.3
Area under the curve of NRS	66.5 ± 27.1	33.4 ± 19.4	<0.01

Data are expressed as mean ± SD, and compared by Mann–Whitney *U* test

No significant differences were detected in nausea or vomiting frequency, degree of pain control satisfaction, dose of diclofenac, or time of first administration of an analgesic after patients exited the operating room. There was no occurrence of local anesthetic toxicity in either group. Hospital stay duration, changes in knee flexion and extension measured by the physiotherapists, and changes in the Knee Society Score were also similar between the groups (Table 3). Comparable results were observed at discharge between the groups. No other side effects or unanticipated postoperative complications were recognized in either group. No participant in either group received intramuscularly injected pentazocine.

**Table 3:** Comparison of postoperative factors or changes between the two groups

	Group L	Group S	*P* value
	(*n* = 16)	(*n* = 17)	
Nausea or vomiting (%)	4/16 (25)	4/17 (24)	0.92
Symptoms of local anesthetic poisoning (%)	0/16 (0)	0/16 (0)	1.00
Satisfaction	4 (1)	4 (1)	0.75
Dose of diclofenac (mg)	0 (25)	25 (25)	0.28
Time until first analgesic usage (hour)	20 (5.4)	18.6 (7.7)	0.59
Hospital stay (days)	25 (7.75)	24 (11.00)	0.69
ROM (extension, temporal change) (degrees)	−8 (11)	−6.9 (10)	0.89
ROM (flexion, temporal change) (degrees)	−6.1 (13)	−6.3 (13)	0.59
KSS (temporal change)	42 (42.5)	49.5 (24.3)	0.86

Data are expressed as the mean (SD), median (interquartile range: value at 75 %–value at 25 %), or n (%) and were compared using the Mann–Whitney *U* test, Wilcoxon signed-rank test, or chi-squared test. Satisfaction was rated using a five-point evaluation system

*ROM* range of motion, *KSS* Knee Society Score

## Discussion

In this study, group S showed superior postoperative pain control to group L within 3–12 h of TKA but not at 0 h. As remifentanil administration was stopped before skin closure, the associated effects had almost disappeared by the time the patients exited the operating room; thus, the dose of LIA was not too low. There was no significant difference at 24 h, which is reasonable, considering that the duration of SNB activity ranges from 8 h to 24 h [19].

There was no difference between the groups in the total analgesics required, time to first analgesic administration, nausea, vomiting, patient satisfaction, or hospital stay duration. The lack of an observed difference may result from FNB alone providing sufficient analgesia, such that the effect of the adjunctive technique is masked. The maximum pain in both groups was still relatively mild, even at 24 h, which supports this explanation. Notably, there are reports challenging the importance of SNB for TKA [20, 21].

The administration method for the local anesthetic mixture was decided by referring to the techniques of Kerr et al. [22]. We did not insert a catheter into the joint because of the risk of infection. Even though the infection risk is low, concerns of infection still exist [23–26].

Incidentally, mobility impairment of the area supplied by the peroneal nerve after plasty of the nerve was reported to occur in approximately 0.3–10 % of TKA cases, irrespective of the presence of nerve block [27]. In our study, one patient in group L experienced a fall on the day after surgery.

There are limitations to our study. First, a better ropivacaine dosage and injection site for the LIA method may be available. An increase in the ropivacaine dose and limitation of the administration area to the posterior site of the knee may lead to different results. Regarding local anesthetic doses, we decided that the total ropivacaine dose for local infiltration should be 200 mg (0.2 % 100 ml), considering that 75 mg (0.375 % 20 ml) of ropivacaine was used as an FNB shot before operation, and approximately 300 mg of ropivacaine was considered to be safe by several papers [10, 28–30]. However, a study similar to our study reached different conclusions [31]. In that study, the doses of FNB and SNB were the same as ours, but the ropivacaine dose for LIA was 300 mg, and injections were only performed in the posterior part of the knee. However, the validity of increasing the ropivacaine dose is controversial [32, 33].

Second, our mixture of local anesthetics did not contain ketorolac or corticosteroids. It was previously reported that inclusion of ketorolac in LIA resulted in reduced pain intensity [34]. Corticosteroids have also been reported to provide effective analgesia [35–39]. Although the possibility of infection exists if administration is performed prior to TKA [40–43], administration during operation as LIA has been reported as safe with regard to infection [35–38]. Devandra et al. demonstrated that there is no difference between SNB and LIA as adjuncts to FNB in terms of postoperative opioid requirement [44]; however, pain scores were higher in the LIA group in the first 12 h. This is compatible with our study, and the periatricular injection did not include ketorolac and corticosteroids as in our study. Thus, the LIA contents of our study should be reconsidered.

## Conclusion

Our results suggest that postoperative pain control within 3–12 h of TKA is better attained through SNB than LIA as a supplement to continuous FNB, without any significant differences in complications, functional outcomes, anesthetic time, or hospitalization. This knowledge will be of value to individuals responsible for determining the anesthesia strategy to control intense postoperative pain after TKA.

## Abbreviations

FNB: Femoral nerve block
SNB: Sciatic nerve block
TKA: Total knee arthroplasty
LIA: Local infiltration analgesia
ASA: American Society of Anesthesiologists
NRS: Numeric Rating Scale
ANOVA: Analysis of variance
NSAID: Nonsteroidal anti-inflammatory drug
SD: Standard deviations
KSS: Knee Society Score
ROM: Range of motion
